# Hemodynamic, renal and hormonal effects of lung protective ventilation during robot-assisted radical prostatectomy, analysis of secondary outcomes from a randomized controlled trial

**DOI:** 10.1186/s12871-021-01401-x

**Published:** 2021-08-05

**Authors:** Sidse Høyer, Frank H. Mose, Peter Ekeløf, Jørgen B. Jensen, Jesper N. Bech

**Affiliations:** 1 University Clinic in Nephrology and Hypertension, Gødstrup Hospital and Aarhus University, 7400 Herning, Denmark; 2 Department of Anesthesiology, Gødstrup Hospital, 7400 Herning, Denmark; 3 Department of Urology, Gødstrup Hospital, 7400 Herning, Denmark; 4 Department of Clinical Medicine, Aarhus University, 8200 Aarhus N, Denmark

**Keywords:** Lung protective ventilation, Robot-assisted radical prostatectomy, Renal function, Renin angiotensin system, Hemodynamics

## Abstract

**Background:**

Lung protective ventilation with low tidal volume (TV) and increased positive end-expiratory pressure (PEEP) can have unfavorable effects on the cardiovascular system. We aimed to investigate whether lung protective ventilation has adverse impact on hemodynamic, renal and hormonal variables.

**Methods:**

In this randomized, single-blinded, placebo-controlled study, 24 patients scheduled for robot-assisted radical prostatectomy were included. Patients were equally randomized to receive either ventilation with a TV of 6 ml/IBW and PEEP of 10 cm H_2_O (LTV-h.PEEP) or ventilation with a TV of 10 ml/IBW and PEEP of 4 cm H_2_O (HTV-l.PEEP). Before, during and after surgery, hemodynamic variables were measured, and blood and urine samples were collected. Blood samples were analyzed for plasma concentrations of electrolytes and vasoactive hormones. Urine samples were analyzed for excretions of electrolytes and markers of nephrotoxicity.

**Results:**

Comparable variables were found among the two groups, except for significantly higher postoperative levels of plasma brain natriuretic peptide (*p* = 0.033), albumin excretion (*p* = 0.012) and excretion of epithelial sodium channel (*p* = 0.045) in the LTV-h.PEEP ventilation group compared to the HTV-l.PEEP ventilation group. In the combined cohort, we found a significant decrease in creatinine clearance (112.0 [83.4;126.7] ml/min at baseline vs. 45.1 [25.4;84.3] ml/min during surgery) and a significant increase in plasma concentrations of renin, angiotensin II, and aldosterone.

**Conclusion:**

Lung protective ventilation was associated with minor adverse hemodynamic and renal effects postoperatively. All patients showed a substantial but transient reduction in renal function accompanied by activation of the renin-angiotensin-aldosterone system.

**Trial registration:**

ClinicalTrials, NCT02551341. Registered 13 September 2015.

**Supplementary Information:**

The online version contains supplementary material available at 10.1186/s12871-021-01401-x.

## Background

Radical prostatectomy is the golden standard for surgical treatment of localized prostate cancer [1]. Compared to open radical prostatectomy (ORP), robot-assisted radical prostatectomy (RARP) is associated with a number of advantages including reduced need of blood transfusion, decreased postoperative pain, shorter hospitalization and fewer complications [2]. RARP is performed under general anesthesia and mechanical ventilation with different values of tidal volume (TV) and positive end-expiratory pressure (PEEP). Recent studies suggest fewer postoperative complications such as pulmonary infections, atelectasis and sepsis when patients are subjected to lung protective ventilation using low TV and high PEEP (LTV-h.PEEP) compared to ventilation using high TV and low PEEP (HTV-l.PEEP) [3, 4]. On the other hand, high PEEP has shown to increase intra-thoracic pressure (ITP) and decrease cardiac output, which causes unfavorable hemodynamic effects [5, 6].

Moreover, RARP requires pneumoperitoneum and Trendelenburg position in order to access the prostate gland during surgery. The increased intra-abdominal pressure (IAP) associated with pneumoperitoneum results in several hemodynamic alterations, including reduced cardiac output and increased vascular resistance due to compression of the abdominal vasculature and compensatory release of stress hormones [7]. This increases the risk of inadequate organ perfusion, which may affect renal function. Animal studies have shown increased serum creatinine and reduced renal blood flow and urine output when pneumoperitoneum is induced [8].

The primary purpose of this randomized study was to evaluate the effect of LTV-h.PEEP ventilation versus HTV-l.PEEP ventilation on PaO_2_ and lung function postoperatively for patients undergoing RARP, which has been reported previously [9]. Secondary variables, such as electrolytes, vasoactive hormones and markers of nephrotoxicity were measured before, during and after surgery. We hypothesized that LTV-h.PEEP ventilation would have adverse effects on hemodynamic, renal and hormonal parameters compared to HTV-l.PEEP ventilation. In this paper we report the effects of ventilation strategy and increased IAP during RARP on the following variables: (1) Hemodynamics: Systolic blood pressure (sBP), diastolic blood pressure (dBP), and heart rate (HR); (2) renal function: Creatinine clearance (CrCl), urinary neutrophil gelatinase-associated lipocalin (u-NGAL), urine aquaporin2 (u-AQP2), and urine epithelial sodium channel (u-ENaC); (3) renal excretions of sodium (u-Na), potassium (u-K), creatinine (u-crea) and albumin (u-alb); (4) plasma concentrations of creatinine (p-crea), sodium (p-Na), potassium (p-K), albumin (p-alb) and vasoactive hormones: P-renin, angiotensin-II (p-angII), aldosterone (p-aldo), vasopressin (p-AVP), and brain natriuretic peptide (p-BNP).

## Methods

### Patients

From September 2015 to January 2017, we recruited 24 patients at the Department of Urology, Regional hospital of West Jutland, Holstebro, Denmark. Included patients were men aged > 18 years and scheduled for RARP. Exclusion criteria were body mass index > 35 kg/m^2^, estimated glomerular filtration rate < 15 ml/min, history of lung disease requiring treatment, severe heart disease classified as New York Heart Association III or above, acute myocardial infarction within the previous year or neuromuscular disease. Withdrawal criteria were complications during surgery i.e. severe bleeding exceeding 1000 ml, saturation below 95% despite increasing fraction of inspired oxygen (FiO_2_) to 0.6, plateau pressure persistently above 30 cm H_2_O, prolonged postoperative procedure due to infection or reoperation, open-surgery conversion or withdrawal of patient consent.

### Design

The study was conducted as a randomized, single-blinded, placebo-controlled study on patients undergoing RARP [9]. Randomization was made by simple envelope method where the clinical coordinator, following randomization allocation, let the involved anesthesiologists know the assignment. The patient and other involved staff and surgeons were blinded for the assignment. Patients were allocated to receive volume-controlled mechanical ventilation with either A) LTV-h.PEEP ventilation: TV of 6 ml/IBW and PEEP of 10 cm H_2_O or B) HTV-l.PEEP ventilation: TV of 10 ml/IBW and PEEP of 4 cm H_2_O. The primary outcomes were PaO_2_ 2 h postoperatively after breathing spontaneously at atmospheric air and postoperative change in lung function, which has been described elsewhere [9]. In this paper we report secondary outcomes of hemodynamics, creatinine clearance (CrCl), renal sodium-, potassium-, albumin-, NGAL-, AQP2- and ENaC- excretions, and plasma levels of electrolytes, RAAS, AVP and BNP in relation to type of ventilation during surgery. The study protocol is in accordance with the CONSORT guidelines.

### Anesthesia, ventilation and surgery

Before surgery all patients received paracetamol 1000 mg, fentanyl 4 microg/kg and a prophylactic dose of cefuroxime 1500 mg and ondansetron 4 mg. Patients were preoxygenated with 100% oxygen for 3 min, after which the anesthesia containing propofol 1–2 mg/kg, remifentanil 2–3 microg/kg and rocuronium 0.5 mg/kg was induced. Anesthesia used for maintenance was propfol 2.2–3.2 microgram/ml, remifentanil 14–20 nanogram/ml and rocuronium 0.2 mg/kg/h. Fluid infusion of Ringer-Acetat 2 ml/kg/hour was maintained during surgery. Any bleeding was replaced with 1–1.5 ml Ringer-Acetat per ml bleeding.

During induction of anesthesia all patients were ventilated with a TV of 10 ml/IBW, a PEEP of 4 cm H_2_O and a FiO_2_ of 0.4. When the patients were considered hemodynamic stable, pneumoperitoneum was induced and the ventilation procedure switched according to allocation. In both groups the respiratory rate was regulated to obtain normoventilation (end-tidal PCO_2_ 5 kPa +/− 0.5 kPa). All patients were placed in 30 ° Trendelenburg during the entire operation. IAP was controlled at 12 mmHg during surgery and increased to 20 mmHg at the end of surgery for vesicourethral anastomosis suturing. By the end of surgery, FiO_2_ was increased to 0.6 and the patient was extubated. After extubation the patient received an oxygen supply of 4 l/min and was transferred to the recovery room. Patients received a suppository of morphine 10 mg, atropinsulfattrituration 5 mg and paraverine 40 mg, and if necessary morphine 5 mg orally or intravenously. Additional anesthetic and surgical procedures have been described previously [9].

### Hemodynamic data

Non-invasive blood pressure and HR were measured preoperatively in the outpatient clinic (Measure 1) and postoperatively at day 1 (Measure 4), day 2 (Measure 5), and day 7 (Measure 6) using standard blood pressure measurement equipment. After induction of anesthesia, blood pressure and HR were measured from an arterial line (Infinity Deltra from Dräger) after 1 h in Trendelenburg (Measure 2) and at the end of operation (Measure 3).

### Blood samples

Blood samples were taken after 30 min’ rest preoperatively at the day of surgery (Blood 1), after 1 h in Trendelenburg perioperatively (Blood 2), at the end of operation (Blood 3), and postoperatively at day 1 (Blood 4), day 2 (Blood 5), and day 7 (Blood 6). P-Na, p-K, p-crea, p-alb, and p-BNP were measured by routine methods at the Department of Clinical Biochemistry. P-renin and p-aldo were measured using immunoradiometric assays, whereas p-angII and p-AVP were measured using radioimmunoassay (RIA) at the University Clinic in Nephrology and Hypertension as previously described [10]. All blood samples were analyzed at Holstebro Hospital, Denmark.

### Urine samples

A 24-h urine sample was collected the day before surgery (Urine 1). At the onset of operation, a urinary catheter was inserted to collect urine during surgery (Urine 2), postoperatively until 8.00 am the next morning (Urine 3), and subsequently 24-h to 8.00 am the day after (Urine 4). U-Na, u-K, u-crea and u-alb were measured by routine procedures at the Department of Clinical Biochemistry. U-AQP2 and u-ENaC were determined by RIA and u-NGAL by ELISA at the University Clinic in Nephrology and Hypertension as previously described [10, 11]. All urine samples were analyzed at Holstebro Hospital, Denmark. CrCl was calculated by the following formula: [u-crea] x urine flow / [p-crea].

### Statistics

Normally distributed data are reported as means with standard deviations (SD); non-normally distributed data are reported as medians with 25–75% quartiles; frequency data are reported as numbers with percentage. *P* values were estimated as two-sided values with a statistical significance level less than 0.05. Data were analyzed according to allocation by intention-to-treat. Single comparisons between groups (LTV-h.PEEP versus HTV-l.PEEP) were analyzed by the unpaired *t* test and the Mann-Whitney *U* test for parametric and non-parametric data, respectively. Multiple comparisons between groups were compared for LTV-h.PEEP group versus HTV-l.PEEP group by a general linear model or a generalized linear model for parametric and non-parametric data, respectively. Comparisons between single means or medians from baseline were analyzed by the paired *t* test for parametric data and the Wilcoxon signed rank test for non-parametric data. Comparisons between several means or medians within groups were analyzed by the ANOVA test or the Friedmann test, respectively. Spearman correlation was used to run univariate correlation analyses for difference in CrCl from baseline to surgery (∆ CrCl). Sample size was determined by power calculation, which has been described previously [9]. Statistical analyses were performed using STATA, version 16.1.

## Results

A total of 24 patients were included; none of the patients meet the prior exclusion criteria. Patients were equally randomized with 12 patients in each group. Comparable baseline characteristics were found among the HTV-l.PEEP and LTV-h.PEEP groups (Table 1). The two groups were similar regarding most perioperative characteristics, including duration of anesthesia (minutes (SD): 225.17 (58.2) and 194.94 (54.0) for HTV-l.PEEP and LTV-h.PEEP, respectively) and duration of surgery (minutes (SD): 180.42 (60.4) and 151.25 (63.6) for HTV-l.PEEP and LTV-h.PEEP, respectively) [9]. However, perioperative blood loss was significantly higher in the HTV-l.PEEP group compared to the LTV-h.PEEP group (medians [25–75% quartiles] of 250 [150; 386] and 110 [100; 150] respectively). None of the patients received blood transfusions.

**Table 1:** Baseline characteristics of 24 men undergoing RARP

Characteristics	Total	HTV-l.PEEP	LTV-h.PEEP	*P*-value
Number	24	12	12	
Age, year^a^	64 (6.6)	64 (6.4)	66 (6.9)	0.526
Height, cm^a^	178 (5)	180 (5)	177 (4)	0.105
Weight, kg^a^	86.2 (10.7)	88.7 (12.9)	83.8 (7.8)	0.270
BMI^a^	27.2 (2.9)	27.4 (3.0)	26.9 (2.8)	0.678
**Office blood pressure**				
sBP, mmHg^a^	152 (17)	149 (16)	156 (19)	0.344
dBP, mmHg^a^	90 (8)	92 (7)	88 (10)	0.294
Pulse^a^	67 (11)	71 (11)	63 (10)	0.092
**Comorbidities**^**b**^				
Hypertension	8 (33.3)	3 (25.0)	5 (41.7)	0.667
Heart disease	1 (4.2)	1 (8.3)	0	1.000
Diabetes mellitus	2 (8.3)	2 (16.7)	0	0.478
Stroke	0	0	0	1.000
**Coexisting conditions**^a^				
Current smoking	3 (12.5)	2 (16.7)	1 (8.3)	1.000
Previous smoking	11 (45.8)	4 (33.3)	7 (58.3)	0.414
Any alcohol intake	20 (83.3)	10 (83.3)	10 (83.3)	1.000
**Risk assessment, preoperative**				
Gleason score^c^	7 (6;7)	7 (7;7)	7 (6;7)	0.870
PSA^a^	8.7 (4.1)	9.9 (4.2)	7.5 (3.9)	0.169
pT stage^c^	7 (7;7)	7 (7;7)	7 (7;7)	1.000

*HTV-l.PEEP* High tidal volume, low positive end-expiratory pressure, *LTV-h.PEEP* Low tidal volume, low positive end-expiratory pressure, *BMI* Body mass index = weight (kg)/height (cm)^2^, *sBP* Systolic blood pressure, *dBP* Diastolic blood pressure, *PSA* Prostate specific antigen, *pT stage* Pathological tumor stage

^a^Parametric data; shown as means ± SD; unpaired *t* test used to test difference between groups

^b^Norminal data; shown as number (%); Fisher’s exact test used to test difference between groups

^c^Non-parametric data; shown as medians [25–75% quartiles]; Mann-Whitney *U* test used to test difference between groups

### Hemodynamics

At day 7 postoperatively, hemodynamic data was collected for approximately half of the patients and due to a large number of missing data, these measurements (Measure 6) were excluded from the analyses (supplementary). Overall, dBP and HR tended to be lower in the LTV-h.PEEP group compared to the HTV-l.PEEP group, though not statistically significant (Table 2).

**Table 2:** Hemodynamic outcomes, stratified according to HTV-l.PEEP versus LTV-h.PEEP

	Measure 1 (Baseline)	Measure 2 (Surgery, 1 h)	Measure 3 (Surgery, end)	Measure 4 (Day 1)	Measure 5 (Day 2)	*P*-value^1^	*P*-value^2^
**sBP (mmHg)**	**152 ± 18**	100 ± 15*	107 ± 17*	128 ± 18*	136 ± 14*	< 0.0001	
HTV-l.PEEP	149 ± 16	96 ± 14*	110 ± 20*	132 ± 22	134 ± 9*	< 0.0001	0.586
LTV-h.PEEP	156 ± 19	104 ± 15*	105 ± 14*	124 ± 13*	139 ± 18*	< 0.0001	
*P*	0.344	0.215	0.511	0.322	0.425		
**dBP (mmHg)**	**90 ± 8**	59 ± 7*	65 ± 13*	74 ± 12*	79 ± 8*	< 0.0001	
HTV-l.PEEP	92 ± 7	61 ± 7*	68 ± 14*	77 ± 15*	81 ± 4*	< 0.0001	0.978
LTV-h.PEEP	88 ± 10	58 ± 7*	62 ± 10*	70 ± 6*	78 ± 10*	< 0.0001	
*P*	0.294	0.290	0.216	0.176	0.428		
**Heart rate (beats/min)**	**67 ± 11**	58 ± 12*	**63 ± 11**	**68 ± 11**	73 ± 9*	< 0.0001	
HTV-l.PEEP	71 ± 11	63 ± 12*	65 ± 11	72 ± 12	73 ± 8*	0.002	0.333
LTV-h.PEEP	63 ± 10	54 ± 10*	60 ± 11	65 ± 9	72 ± 11*	< 0.0001	
*P*	0.092	0.081	0.313	0.122	0.721		
**Pulse pressure (mmHg)**	**62 ± 19**	41 ± 13*	**67 ± 20**	54 ± 13*	**57 ± 13**	< 0.0001	
HTV-l.PEEP	57 ± 14	36 ± 10*	61 ± 17	55 ± 12	53 ± 10	0.0009	0.704
LTV-h.PEEP	68 ± 22	47 ± 13*	73 ± 22	54 ± 15*	61 ± 15	0.0002	
*P*	0.170	0.042	0.150	0.868	0.171		

*HTV-l.PEEP* High tidal volume, low positive end-expiratory pressure, *LTV-h.PEEP* Low tidal volume, low positive end-expiratory pressure, *sBP* Systolic blood pressure, *dBP* Diastolic blood pressure, *sBP* Systolic blood pressure, *dBP* Diastolic blood pressure

All data are considered normally distributed; shown as means ± SD; paired *t* test used to test difference from baseline, * = *p* < 0.05; unpaired *t* test used to test difference between groups

^1^*P*-value showing the difference in means within groups analyzed by repeated measure ANOVA test

^2^*P*-value showing the difference in means between groups analyzed by general linear model

**Fig. 1:**
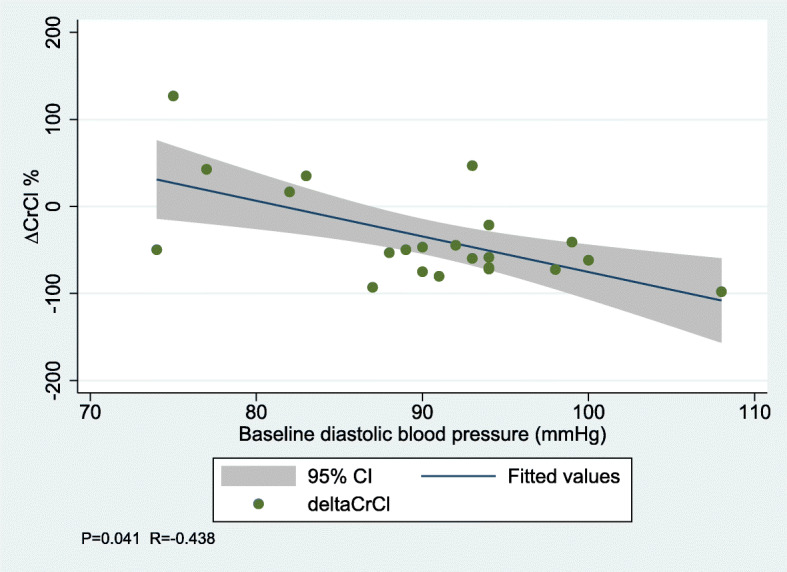
Correlation between baseline diastolic blood pressure and change in creatinine clearance

**Fig. 2:**
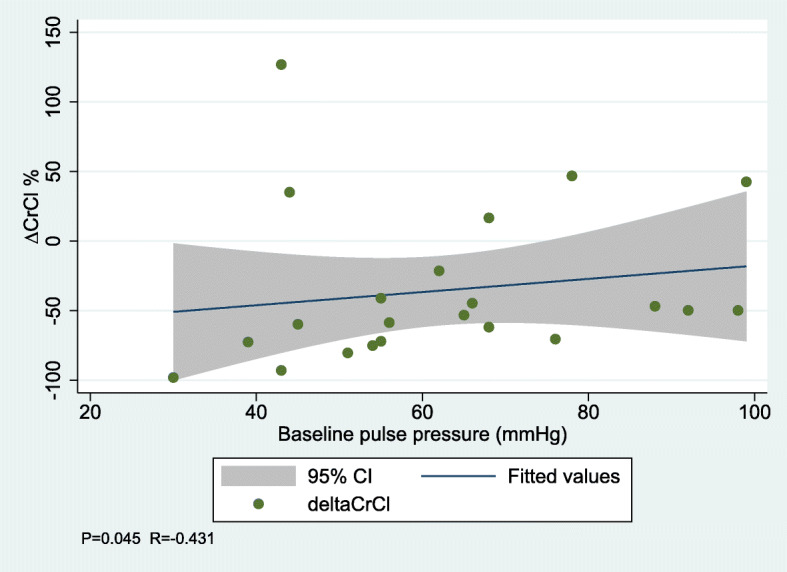
Correlation between baseline pulse pressure and change in creatinine clearance

Through all measurements in both groups combined, sBP and dBP were significantly lower with a marked decrease during surgery compared to baseline levels. HR decreased during anesthesia, but in reverse increased at day 2 postoperatively. In correlation analyses, increased baseline dBP and decreased baseline pulse pressure were associated with a decline in ∆ CrCl (Table 3, Figs. 1 and 2).

**Table 3:** Correlation between baseline clinical outcomes, fluid infusion and need of efedrin and difference in creatinine clearance (∆ CrCl) by spearman coefficient analysis

	∆ CrCl	
	*P*-value	R-value
sBP	0.180	0.297
dBP	0.041	−0.438
Pulse pressure	0.045	0.431
Creatinine clearance	0.434	−0.176
P-Renin	0.840	0.046
P-Angiotensin-II	0.134	0.330
P-Aldosterone	0.106	0.354
P-Vasopressin	0.093	−0.367
P-BNP	0.306	0.235
U-albumin	0.662	0.099
UACR	0.324	0.221
U-NGAL	0.839	0.046
IV fluid infusion	0.225	0.270
Need of efedrin	0.825	−0.050

*sBP* Systolic blood pressure, *dBP* Diastolic blood pressure, *P-BNP* Plasma concentrations of brain natriuretic peptide, *UACR* Urine albumin creatinine ratio, *U-NGAL* urinary neutrophil gelatinase-associated lipocalin

### Renal function and electrolytes

Overall, changes in renal function, electrolytes and markers of nephrotoxicity were similar between the LTV-h.PEEP group and the HTV-l.PEEP group. However, some differences were found between groups. We found a significantly lower p-K from the end of surgery until day 2 postoperatively and higher creatinine adjusted urine albumin (UACR) and u-ENaC (u-ENaC_Cr_) at day 7 postoperatively were seen in the LTV-h.PEEP group compared to the HTV-l.PEEP group (Tables 4, 5 and 6). When analyzing the difference in several means or medians by the general or generalized linear model for these measurements, no significant difference was observed between the two groups.

**Table 4:** Blood samples, stratified according to HTV-l.PEEP versus LTV-h.PEEP

	Blood 1 (Baseline)	Blood 2 (Surgery, 1 h)	Blood 3 (Surgery, end)	Blood 4 (Day 1)	Blood 5 (Day 2)	Blood 6 (Day 7)	*P*-value^1^	*P*-value^2^
**P-sodium (mmol/l)**	**140 ± 3**	139 ± 3*	139 ± 3*	138 ± 3*	139 ± 3*	**137 ± 8**	0.113	
HTV-l.PEEP	140 ± 1	139 ± 1*	139 ± 1*	139 ± 2*	140 ± 1	140 ± 2	0.022	0.062
LTV-h.PEEP	139 ± 4	139 ± 4	139 ± 4	137 ± 4*	138 ± 3*	135 ± 10	0.154	
*P*	0.310	0.557	0.518	0.239	0.053	0.124		
**P-potassium (mmol/l)**	**4.0 ± 0.3**	4.2 ± 0.4*	4.3 ± 0.4*	**3.9 ± 0.2**	**4.0 ± 0.3**	**4.0 ± 0.2**	< 0.0001	
HTV-l.PEEP	4.1 ± 0.3	4.4 ± 0.3*	4.5 ± 0.3*	4.1 ± 0.2	4.2 ± 0.2	4.1 ± 0.3	< 0.0001	0.889
LTV-h.PEEP	3.9 ± 0.3	4.1 ± 0.5	4.2 ± 0.4*	3.8 ± 0.2	3.9 ± 0.2	4.0 ± 0.2	0.0004	
*P*	0.125	0.126	0.042	0.0009	0.002	0.134		
**P-albumin (g/l)**	**40 ± 2**	36 ± 2*	34 ± 2*	35 ± 2*	36 ± 2*	38 ± 3*	< 0.0001	
HTV-l.PEEP	40 ± 2	35 ± 2*	34 ± 2*	35 ± 1*	37 ± 2*	38 ± 3	< 0.0001	0.131
LTV-h.PEEP	40 ± 2	36 ± 2*	35 ± 2*	35 ± 2*	36 ± 3*	37 ± 3*	< 0.0001	
*P*	0.543	0.189	0.392	0.602	0.207	0.470		
**P-creatinine (μmol/l)**	**84 ± 10**	**83 ± 12**	90 ± 12*	**83 ± 12**	**83 ± 11**	82 ± 10*	0.0001	
HTV-l.PEEP	85 ± 9	83 ± 8	91 ± 10*	83 ± 13	82 ± 8	82 ± 10	0.001	0.773
LTV-h.PEEP	83 ± 11	84 ± 16	89 ± 15	82 ± 11	85 ± 14	81 ± 11	0.054	
*P*	0.551	0.858	0.835	0.857	0.525	0.728		

*HTV-l.PEEP* High tidal volume, low positive end-expiratory pressure, *LTV-h.PEEP* Low tidal volume, low positive end-expiratory pressure

All data are considered normally distributed; shown as means ± SD; paired *t* test used to test difference from baseline, * = *p* < 0.05; unpaired *t* test used to test difference between groups

^1^*P*-value showing the difference in means within groups analyzed by repeated measure ANOVA test

^2^*P*-value showing the difference in means between groups analyzed by general linear model

**Table 5:** Urine samples, stratified according to HTV-l.PEEP versus LTV-h.PEEP

	Urine 1 (Baseline)	Urine 2 (Surgery)	Urine 3 (Day 1)	Urine 4 (Day 2)	*P*-value^1^	*P*-value^2^
**FeNa (%)**	**0.83 ± 0.30**	**0.83 ± 0.63**	**0.88 ± 0.34**	0.58 ± 0.34*	0.038	
HTV-l.PEEP	0.84 ± 0.27	0.88 ± 0.55	0.89 ± 0.37	0.60 ± 0.36*	0.237	0.939
LTV-h.PEEP	0.81 ± 0.33	0.78 ± 0.71	0.87 ± 0.32	0.56 ± 0.32*	0.255	
*P*	0.807	0.705	0.909	0.787		
**FeK (%)**	**11.0 ± 3.2**	**12.3 ± 5.6**	13.4 ± 4.0*	8.8 ± 3.6*	0.0004	
HTV-l.PEEP	11.1 ± 3.3	12.6 ± 5.8	12.8 ± 4.2	8.2 ± 3.1*	0.011	0.475
LTV-h.PEEP	10.9 ± 3.2	12.0 ± 5.7	14.0 ± 3.8*	9.4 ± 4.2	0.047	
*P*	0.883	0.793	0.482	0.444		
**Creatinine clearance (ml/min)**^***3***^	**112.0 [83.4;126.7]**	45.1 [25.4;84.3]*	**112.0 [92.0; 134.2]**	122.0 [106.4;134.0]*	< 0.0001	
HTV-l.PEEP	113.7 [81.3;131.2]	49.0 [32.0;75.1]*	117.8 [93.2;145.1]	123.2 [117.5;136.9]*	0.0004	0.572
LTV-h.PEEP	112.0 [83.4;117.7]	33.3 [25.4;147.3]	103.5 [92.4;131.9]	121.4 [104.9;133.7]*	0.034	
*P*	0.722	0.833	0.514	0.525		
**UACR (mmol/mmol crea)**	**0.7 [0.5;1.4]**	11.3 [8.0;24.3]*	15.8 [12.9;19.6]*	12.8 [10.1;18.7]*	< 0.0001	
HTV-l.PEEP	0.8 [0.5;1.3]	13.0 [6.8;22.7]*	13.1 [7.3;18.5]*	10.7 [7.7;13.3]*	0.0002	0.312
LTV-h.PEEP	0.7 [0.4;1.1]	11.2 [9.6;28.6]*	17.4 [15.0;20.3]*	16.4 [12.8;24.6]*	< 0.0001	
*P*	0.630	0.671	0.089	0.012		

*HTV-l.PEEP* High tidal volume, low positive end-expiratory pressure, *LTV-h.PEEP* Low tidal volume, low positive end-expiratory pressure, *FeNa* Fractional excretion of sodium, *FeK* Fractional excretion of potassium, *UAER* Urine albumin excretion rate, *UACR* Urine albumin creatinine ratio

Data considered normally distributed; shown as means ± SD; paired *t* test used to test difference from baseline, * = *p* < 0.05; unpaired *t* test used to test difference between groups

Data considered non-normally distributes; shown as medians [25–75% quartiles]; Wilcoxon signed rank test used to test difference from baseline, * = *p* < 0.05; Mann-Whitney *U* test used to test difference between groups

^1^*P*-value showing the difference in means or medians within groups analyzed by repeated measure ANOVA test or Friedmann test, respectively

^2^*P*-value showing the difference in means or medians between groups analyzed by general linear model or generalized linear model, respectively

^3^Calculated using the CKD-EPIcrea formula

**Table 6:** Creatinine adjusted urine samples, stratified according to HTV-l.PEEP versus LTV-h.PEEP

	Urine 1 (Baseline)	Urine 2 (Surgery)	Urine 3 (Day 1)	Urine 4 (Day 2)	*P*-value^1^	*P*-value^1^
**U-AQP2**_**Cr**_ **(ng/mmol crea)**	**155.3 [129.8;186.6]**	**153.8 [119.8;186.9]**	**185.7 [164.5;203.1]**	**149.2 [120.7;182.2]**	0.021	
HTV-l.PEEP	165.9 [132.4;259.1]	171.2 [127.1;197.7]	192.7 [151.5;222.0]	149.2 [115.6;167.5]*	0.069	0.090
LTV-h.PEEP	143.7 [119.8;175.0]	139.0 [109.3;180.3]	184.0 [174.8;188.4]*	152.0 [120.7;225.5]	0.004	
*P*	0.198	0.291	1.000	0.551		
**U-ENaC**_**Cr**_ **(ng/mmol crea)**	**87.5 [63.9;138.0]**	160.0 [102.3;244.0]*	106.2 [80.2;129.7]*	121.2 [104.8;150.2]*	< 0.0001	
HTV-l.PEEP	79.8 [57.0;123.5]	160.0 [110.9;259.3]*	96.1 [73.7;111.9]	108.3 [101.6;132.1]	0.005	0.627
LTV-h.PEEP	88.8 [80.4;143.0]	168.4 [102.3;240.6]*	113.9 [98.7;155.1]*	130.5 [117.0;167.9]*	0.002	
*P*	0.198	0.843	0.089	0.045		
**U-NGAL**_**Cr**_ **(μg/mmol crea)**	**1.0 [0.8;2.2]**	8.9 [5.1;21.9]*	1.0 [0.7;2.0]*	**1.2 [0.8;2.0]**	< 0.0001	
HTV-l.PEEP	1.0 [0.8;2.2]	10.6 [6.8;21.9]*	1.4 [0.7;9.9]	1.1 [0.8;1.5]	0.0004	0.798
LTV-h.PEEP	1.0 [0.7;2.7]	8.3 [3.8;20.9]*	1.0 [0.6;1,5]*	1.5 [1.0;2.4]	0.0002	
*P*	0.810	0.590	0.178	0.347		

*HTV-l.PEEP* High tidal volume, low positive end-expiratory pressure, *LTV-h.PEEP* Low tidal volume, low positive end-expiratory pressure, *U-AQP2*_*cr*_ creatinine adjusted urinary aquaporin2, *U-ENaC*_*Cr*_ creatinine adjusted urinary epithelial sodium channel, *U-NGAL*_*Cr*_ creatinine adjusted urinary neutrophil gelatinase-associated lipocalin

All data considered non-normally distributes; shown as medians [25–75% quartiles]; Wilcoxon signed rank test used to test difference from baseline, * = *p* < 0.05; Mann-Whitney *U* test used to test difference between groups

^1^*P*-value showing the difference in medians within groups analyzed by Friedmann test

^2^*P*-value showing the difference in medians between groups analyzed by generalized linear model

In both groups combined, CrCl decreased significantly from 112.0 ml/min at baseline to 45.1 ml/min during surgery, while the patients had increased IAP in Trendelenburg position (*p* = 0.006) (Table 5). At day 1 postoperatively CrCl was returned to normal. Also, UACR increased significantly during surgery and even more pronouncedly at day 1 and day 2 postoperatively. Similarly, a decline in p-alb was measured during surgery until day 7 postoperatively. Though some statistical significance in single values was seen for p-Na, p-K, p-crea and the fractional excretion of sodium (FeNa) and potassium (FeK), the results were inconsistent across measurements. Furthermore, creatinine adjusted u-NGAL (u-NGAL_Cr_) increased significantly during surgery but quickly normalized at day 1 postoperatively. Also, u-ENaC_Cr_ increased significantly during surgery and at day 1 and 2 postoperatively, whereas the creatinine adjusted u-AQP2 (u-AQP2_Cr_) showed no considerable change across measurements.

**Table 7:** Blood samples, stratified according to HTV-l.PEEP versus LTV-h.PEEP

	Blood 1 (Baseline)	Blood 2 (Surgery, 1 h)	Blood 3 (Surgery, end)	Blood 4 (Day 1)	Blood 5 (Day 2)	Blood 6 (Day 7)	*P*-value^1^	*P*-value^2^
**P-renin (pg/ml)**	**9 [5;17]**	39 [15; 66]*	48 [15;78]*	13 [8.0;19]*	**8 [6;16]**	**11 [5;26]**	< 0.0001	
HTV-l.PEEP	9 [6; 14]	44 [18;60]*	46 [15;58]*	12 [8;16]*	8 [6;15]	11 [4;23]	< 0.0001	0.821
LTV-h.PEEP	9 [5;20]	34 [13;70]*	57 [16;115]*	13 [9;30]	8 [6;16]	10 [5;44]	< 0.0001	
*P*	0.640	0.899	0.326	0.659	0.723	0.600		
**P-angiotensin II (pg/ml)**	**5 [4;7]**	34 [11;74]*	34 [15;87]*	8 [5;13]*	**6 [4;7]**	5 [4;15]*	< 0.0001	
HTV-l.PEEP	5 [4;8]	42 [17;69]*	46 [15;68]*	10 [6;14]*	7 [5;7]	10 [4;15]	< 0.0001	0.993
LTV-h.PEEP	5 [3;7]	14 [9;79]*	25 [16;117]*	7 [5;10]*	4 [4;7]	5 [4;13]	< 0.0001	
*P*	0.373	0.370	0.681	0.338	0.247	0.677		
**P-aldosterone (pmol/l)**	**250 [196;320]**	457 [344;527]*	433 [256;557]*	**220 [169;295]**	**222 [178;269]**	**251 [204;357]**	< 0.0001	
HTV-l.PEEP	220 [185;279]	417 [289;500]*	329 [229.;467]*	221 [169;273]	219 [178;249]	222 [204;279]	0.014	0.425
LTV-h.PEEP	284 [223;374]	476 [374;570]*	516 [313;603]*	218 [177;310]*	229 [176;292]	301 [213;397]	0.003	
*P*	0.097	0.347	0.128	0.966	0.767	0.164		
**P-vasopressin (pg/ml)**	**0.3 [0.2;0.3]**	0.3 [0.3;0.4]*	0.4 [0.3;0.5]*	0.4 [0.3;0.5]*	0.4 [0.3;0.6]*	**0.3 [0.3;0.3]**	0.0001	
HTV-l.PEEP	0.3 [0.2;0.3]	0.3 [0.3;0.5]*	0.4 [0.3;0.6]*	0.4 [0.4;0.5]*	0.4 [0.3;0.6]*	0.3 [0.3;0.4]*	0.0004	0.999
LTV-h.PEEP	0.3 [0.2;0.3]	0.3 [0.3;0.3]	0.3 [0.2;0.4]	0.3 [0.3;0.5]	0.3 [0.3;0.5]	0.3 [0.2;0.3]	0.099	
*P*	0.947	0.473	0.127	0.138	0.315	0.045		
**P-brain natriuretic peptide (pmol/l)**	**5.4 [2.9;10.8]**	6.6 [3.6;10.6]*	7.6 [4.3;11.3]*	7.8 [4.3;13.1]*	**5.9 [4.2;10.7]**	**4.3 [2.9;9.0]**	0.001	
HTV-l.PEEP	5.4 [2.9;11.1]	6.9 [3.5;10.6]*	8.0 [4.4;11.3]*	9.5 [3.0;12.0]	4.2 [2.9;7.15]	3.2 [2.9;4.2]	0.003	0.319
LTV-h.PEEP	4.8 [3.3;8.5]	6.2 [3.7;12.9]*	7.3 [3.9;11.9]*	7.3 [5.2;16.5]*	8.1 [5.8;14.3]*	7.2 [4.5;13.5]	0.030	
*P*	0.845	0.811	0.854	0.712	0.018	0.033		

*HTV-l.PEEP* High tidal volume, low positive end-expiratory pressure, *LTV-h.PEEP* Low tidal volume, low positive end-expiratory pressure

All data considered non-normally distributes; shown as medians [25–75% quartiles]; Wilcoxon signed rank test used to test difference from baseline, * = *p* < 0.05; Mann-Whitney *U* test used to test difference between groups

^1^*P*-value showing the difference in medians within groups analyzed by Friedmann test

^2^*P*-value showing the difference in medians between groups analyzed by generalized linear model

### Vasoactive hormones

There was no difference in RAAS between the LTV-h.PEEP and HTV-l.PEEP groups. The LTV-h.PEEP group had significantly higher p-BNP at day 2 and day 7 postoperatively compared to the HTV-l.PEEP group (Table 7).

For the total cohort, p-renin, p-angII, p-aldo, p-AVP and p-BNP all significantly increased during surgery compared to baseline levels. P-aldo was quickly normalized at day 1 postoperatively, whereas p-renin, p-angII and p-BNP were normalized at day 2 postoperatively and p-AVP at day 7 postoperatively.

## Discussion

In this report, only minor hemodynamic, renal and hormonal differences were observed for patients subjected to LTV-h.PEEP ventilation compared to HTV-l.PEEP ventilation. The concept of LTV-h.PEEP ventilation is to reduce the TV to 6–8 ml/IBW in combination with a moderate to high PEEP of 8–10 cm H_2_O, which is in contrast to formerly used conditional ventilation using TV of 8–10 ml/IBW and a low to modest PEEP of 0–4 cm H_2_O. In this study, all patients allocated to receive LTV-h.PEEP ventilation were ventilated with a TV of 6 ml/IBW and a PEEP of 10 cm H_2_O. LTV-h.PEEP ventilation was associated with higher postoperative values of p-BNP, UACR and u-ENaC_Cr_ compared to HTV-l.PEEP ventilation and this finding has to our knowledge not been reported elsewhere. The increased values of p-BNP, UACR and u-ENaC_Cr_ may be explained by altered pressure conditions in the intra-thoracic and intra-abdominal cavities, causing prolonged hemodynamic or renal stress. An increased PEEP and an induction of pneumoperitoneum are known to cause adverse cardiac vascular effects such as increased afterload and reduced cardiac output. On the other hand, the Trendelenburg position favors the cardiovascular system by increasing venous return and subsequently cardiac output [12]. Thus, the exact physiological consequences of ventilation strategy and surgical interventions are complex and remain unclear. Blood loss during surgery was higher for patients ventilated with HTV-l.PEEP compared to patients ventilated with LTV-h.PEEP. Conversely, perioperative fluid replacement corresponds to the blood loss in each group and thus, the difference between the two groups is unlikely of clinical significance. Also, intraoperative bleeding has not been associated with type of ventilation during surgery [13].

In concordance with our findings, Cortjens et al. investigated whether the type of mechanical ventilation was associated with development of acute kidney injury (AKI). In a cohort of patients without acute lung injury they found no beneficial effects of ventilation with low tidal volume (6 mL/kg) compared to high tidal volume (10 mL/kg) [14]. In contrast, another clinical study of patients with acute lung injury found reduced risk of AKI with low tidal volume ventilation compared to high tidal volume ventilation [15]. However, both studies evaluated the effect of TV on AKI and not the effect of PEEP as in our study.

For both cohorts combined, a significant decrease in CrCl associated with a substantial activation of RAAS was seen in relation to RARP. Furthermore, u-NGAL_Cr_ and UACR increased, indicating renal tubular and glomerular injury, respectively. Also, the minor increase in p-BNP suggested some wall stress of the cardiac myocytes, probably due to the altered ITP despite of the anesthesia-induced decrease in sBP and dBP. In total, these findings suggest considerable hemodynamic, renal, and hormonal alterations associated with the RARP procedure. The mechanisms resulting in these effects are likely multifactorial. The hemodynamic, renal and hormonal outcomes of RARP may be affected by several factors, including the level of IAP, degree of Trendelenburg position, baseline volume status, insufflated CO_2_, oxidative stress, and other hormonal factors i.e. endothelin and NO [7, 16, 17].

Few other studies investigated renal and hormonal outcomes for patients undergoing radical prostatectomy. Similar to our study, Kancir et al. found a perioperative decrease in CrCl and an increase in u-alb that remained elevated at day 14 postoperatively for patients undergoing ORP. Also, Kancir et al. reported activation of RAAS, but not as substantial as our findings, which may be explained by the procedure being ORP and not RARP as in our study. In contrast to our study, they reported elevated u-NGAL at discharge but not during surgery [18]. However, other studies reported u-NGAL to be an early biomarker (2–6 h after surgery) of tubular damage, which support our findings [19, 20]. In another RARP study, Islamoglu et al. found significant increased p-crea postoperatively, but no statistical significant change in eGFR or renal artery resistance measured by Doppler ultrasonography. Since measurements were performed 24 h after RARP they were not able to make conclusions for the perioperative period [21]. Also other studies that evaluated the effects of pneumoperitoneum on hormonal alterations support our findings of RAAS activation. Though the anesthesia by itself is known to reduce sympathetic tone and thereby decrease effective intravascular volume that causes activation of vasoactive hormones, it has been reported that pneumoperitoneum independently stimulates RAAS and increases p-AVP [22, 23]. A porcine study demonstrated a positive correlation between IAP and the levels of renin and aldosterone with a significant increase in these hormones starting at an IAP of 15 mmHg. In addition, they reported quick normalization in renin and aldosterone levels after abdominal decompression [24]. In our study, IAP was continuously regulated aiming at values of approx. 12 mmHg.

Interestingly, higher baseline dBP was associated with an aggravated decrease in ∆ CrCl compared to men with lower dBP at baseline. Also, lower baseline pulse pressure was associated with an aggravated decrease in ∆ CrCl, which likely reflects an isolated elevation of the baseline dBP. These findings suggest that the decrease in renal function observed during surgery, at least partly could be explained by hemodynamic mechanisms such as the presence of diastolic hypertension. In contrast to our findings, Jin et al. found similar baseline dBP in patients developing AKI and patients who did not develop AKI after cardiac surgery. Also, they reported nadir values of postoperative diastolic perfusion pressure (dBP – central venous pressure) to be associated with the development of AKI [25].

### Strengths and limitations

A major strength in this study is a systematic blood and urine sample collection before, during and after surgery, which improves the evidence of chronological measures for an entire RARP procedure. Though we drew blood samples 1 week after surgery, urine samples were only collected until day 2 postoperatively and thus no long-term effects were examined.

Like other previous studies, we chose a pragmatic approach by using fixed levels of PEEP [26]. Another approach is to use individualized PEEP, which potentially leads to more homogeneous results [27, 28].

Another limitation of this study is the small sample size, which may have been inadequate to reveal differences between the two types of ventilation or properly estimate hemodynamic, renal and hormonal variables. Also, our cohort consisted of 24 relatively young and healthy men, who physiologically may compensate sufficiently to the effects of RARP due to better hemodynamic tolerance and renal adjustment. In a cohort of elderly men with more comorbidity the effects of RARP or ventilation strategy may be more prolonged and clinically significant. Also, our findings may only apply to patients with BMI < 35, since obese patients with BMI > 35 in Trendelenburg are more likely to develop pulmonary and extra-pulmonary complications such as renal impairment [26]. We did not obtain information on use of medication e.g. RAAS inhibitors, which may interact with the measured outcomes. In our cohort, few patients were diagnosed with hypertension, heart disease and diabetes mellitus and thus likely to have any RAAS inhibitor prescribed. However, an evaluation of the use of RAAS inhibitors in relation to hemodynamic, renal and hormonal alterations for patients undergoing RARP would be interesting for further investigation.

## Conclusion

Our study indicated minor adverse renal and hemodynamic effects of LTV-h.PEEP ventilation compared to HTV-l.PEEP ventilation for patients undergoing RARP. Ventilation strategy did not seem to have hormonal consequences. However, in the combined cohort we found a profound, but transient decrease in CrCl accompanied by RAAS activation when IAP was elevated to 12 mmHg and patients were positioned in 30-degree Trendelenburg.

## Supplementary Information

**Additional file 1 Table S2.** Hemodynamic outcomes – day 7 postoperatively.

## Data Availability

The data associated with the paper are not publicly available due to its content of confidential personal health related information.

## Abbreviations

AKI: Acute kidney injury
CrCl: Creatinine clearance
dBP: Diastolic blood pressure
FeK: Fractional excretion of potassium
FeNa: Fractional excretion of sodium
FiO_2_: Fraction of inspired oxygen
HR: Heart rate
HTV-l.PEEP: High tidal volume, low positive end-expiratory pressure
IAP: Intra-abdominal pressure
ITP: Intra-thoracic pressure
LTV-h.PEEP: Low tidal volume, high positive end-expiratory pressure
ORP: Open radical prostatectomy
p-alb: Plasma concentrations of albumin
p-aldo: Plasma concentrations of aldosterone
p-angII: Plasma concentrations of angiotensin II
p-AVP: Plasma concentrations of vasopressin
p-BNP: Plasma concentrations of brain natriuretic peptide
p-crea: Plasma concentrations of creatinine
PEEP: Positive end-expiratory pressure
p-K: Plasma concentrations of potassium
p-Na: Plasma concentrations of sodium
RAAS: Renin-angiotensin-aldosterone system
RARP: Robot-assisted radical prostatectomy
RIA: Radioimmunoassay
sBP: Systolic blood pressure
TV: Tidal volume
UACR: Creatinine adjusted urine albumin
u-alb: Renal excretions of albumin
u-AQP2: Urinary aquaporin2
u-AQP2_Cr_: Creatinine adjusted urinary aquaporin2
u-crea: Renal excretions of creatinine
u-ENaC: Urinary epithelial sodium channel
u-ENaC_Cr_: Creatinine adjusted urinary epithelial sodium channel
u-K: Renal excretions of potassium
u-Na: Renal excretions of sodium
u-NGAL: Urinary neutrophil gelatinase-associated lipocalin
u-NGAL_Cr_: Creatinine adjusted urinary neutrophil gelatinase-associated lipocalin
∆CrCl: Difference in CrCl from baseline to surgery
