# Supplementary material for: Hemodynamic, renal and hormonal effects of lung protective ventilation during robot-assisted radical prostatectomy, analysis of secondary outcomes from a randomized controlled trial
Source: BMC Anesthesiol. 2021 Aug 5;21:200. doi: 10.1186/s12871-021-01401-x (PMC8340542; doi:10.1186/s12871-021-01401-x)
Supplement: Supplementary file 1 — Additional file 1 Table S2. Hemodynamic outcomes – day 7 postoperatively. [file 12871_2021_1401_MOESM1_ESM.docx]

**SUPLEMENTARY**

**Table 2 – Hemodynamic outcomes – day 7 postoperatively**

|  | **sBP (mmHg)** | **dBP (mmHg)** | **Heart rate (beats/min)** | **Pulse pressure (mmHg)** |
| --- | --- | --- | --- | --- |
| HTV-l.PEEP | 139.0 (12.2) | 82.6 (8.9) | 63.3 (9.6) | 56.4 (7.2) |
| Missings (%) | 4 (33.3) | 4 (33.3) | 4 (33.3) | 4 (33.3) |
| LTV-h.PEEP | 128.2 (10.4) | 73.2 (6.4) | 56.4 (7.2) | 55.0 (10.0) |
| Missings (%) | 6 (50.0) | 6 (50.0) | 5 (41.7) | 6 (50.0) |

HTV-l.PEEP: High tidal volume, low positive end-expiratory pressure. LTV-h.PEEP: Low tidal volume, low positive end-expiratory pressure. sBP: Systolic blood pressure. dBP: Diastolic blood pressure. sBP: Systolic blood pressure. dBP: Diastolic blood pressure.
